# P-1915. Factors Associated with Post-discharge Mortality among SARS-CoV-2-infected Adult Patients with Severe Acute Respiratory Infection in Bangladesh during 2020–2023

**DOI:** 10.1093/ofid/ofae631.2075

**Published:** 2025-01-29

**Authors:** Saju Bhuiya, Md Ariful Islam, Tanzir Ahmed Shuvo, Md Abdul Aleem, Md Zakiul Hassan, Fahmida Chowdhury

**Affiliations:** icddr,b, Dhaka, Dhaka, Bangladesh; icddr,b, Dhaka, Dhaka, Bangladesh; icddr,b, Dhaka, Dhaka, Bangladesh; icddr,b, Dhaka, Dhaka, Bangladesh; icddr,b, Dhaka, Dhaka, Bangladesh; icddr,b, Dhaka, Dhaka, Bangladesh

## Abstract

**Background:**

To better understand the true mortality burden among SARS-CoV-2-infected patients with severe respiratory illnesses, in resource-limited settings in Bangladesh, it is crucial to consider post-discharge deaths along with in-hospital deaths. We estimated the proportion of SARS-CoV-2-associated adult SARI patients who died after discharge and identified factors associated with these deaths.

**Methods:**

From March 2020–December 2023, we analyzed WHO defined severe acute respiratory infection (SARI) patient data from nine tertiary-level hospitals participating in national hospital-based influenza surveillance in Bangladesh. We estimated the proportion of SARS-CoV-2-associated deaths that occurred within 30 days after hospital-discharge and compared demographics, clinical characteristics between SARS-CoV-2-associated decedents and SARS-CoV-2 survivors. Logistic regression analyses were performed to determine for factors associated with SARS-CoV-2-infected post-discharge deaths.

**Results:**

We identified 7,816 SARI patients with a median age of 50 years (IQR: 32–60), 62% were male, and 16.4% (1,280) were laboratory-confirmed SARS-CoV-2-infected patients. Of the SARS-CoV-2-infected patients, 9.8% (126) died during their hospital stay. Of 1,154 patients who were alive at discharge, we followed 1,108 (96%) patients, and 111 (10%) died within 30 days after discharge. Patients aged 40-60 (aOR: 8.3, 95% CI: 2.45-27.70) and those aged ≥60 (aOR: 26.3, 95% CI: 8.01-86.2) compared to 18-40 years, residents of rural areas (aOR: 1.91, 95% CI: 1.10-3.31), and individuals with difficulty breathing (aOR: 4.13, 95% CI: 1.75-9.75) or kidney diseases (aOR: 4.62, 95% CI: 1.32-16.14) were significantly associated with a higher risk of post-discharge death.

**Conclusion:**

Almost half of the deaths among SARS-CoV-2-infected SARI patients in Bangladesh occurred within 30 days post-discharge. To improve post-discharge recovery among vulnerable patients with potential risk attributes, hospitals in resource-limited settings could develop safe-discharge algorithms, strengthen post-discharge care plans, and establish outpatient monitoring of recently discharged patients.

**Disclosures:**

All Authors: No reported disclosures

